# The physical basis of fabrication of amyloid-based hydrogels by lysozyme

**DOI:** 10.1039/c9ra07179b

**Published:** 2019-11-15

**Authors:** Anumita Kumari, Basir Ahmad

**Affiliations:** School of Chemical Sciences, UM-DAE Centre for Excellence in Basic Sciences, University of Mumbai Mumbai-400098 India; Protein Assembly Laboratory, JH-Institute of Molecular Medicine, Jamia Hamdard Hamdard Nagar New Delhi-110062 India ahmad.basir1@gmail.com +918452897617; School of Chemical Sciences, UM-DAE Centre for Excellence in Basic Sciences, University of Mumbai Mumbai-400098 India

## Abstract

The fabrication of amyloid-based hydrogels has attracted remarkable attention within the field of materials science and technology. These materials have a multitude of potential applications in the biomaterials field such as developing scaffolds for tissue engineering, drug delivery and hygiene products. Despite the potential new applications of these materials, the physical nature of their assembly is not well understood. In this study, we have investigated how the conformation of the amyloid precursor state (I) is formed and correlated with the assembly of amyloid-based hydrogels. A transparent hydrogel was fabricated at pH 7.4 by cooling of the temperature-induced unfolded state of hen egg white lysozyme (HEWL). The completely unfolded state (U) at the gelation concentration of HEWL was obtained around 90 °C in the presence of tris(2-carboxyethyl)phosphine (TCEP), with a TCEP/HEWL molar ratio of 4 : 1. The characterization of the hydrogel showed that it was composed of an amyloid fibril-like material. The physical nature of its assembly was examined in detail and it was found that the hydrogel formation reaction was a three-step, four-states process (U → I → F → H). We concluded that the properties of the pre-molten globule state (I) of the protein correlated only with the fibrillation process, whereas the assembly of the fibrils into an hydrogel was found to be almost independent of the I state. Thus, the study presented here provides a complete biophysical insight into the pathway of lysozyme hydrogel assembly.

## Abstract 2

Schematic of heating- and cooling-induced transitions between HEWL states, and the subsequent formation of the hydrogel.

## Graphical abstract

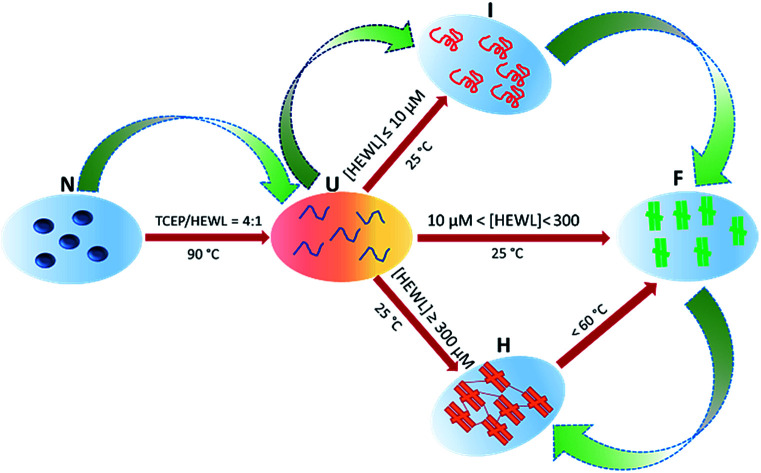

## 1. Introduction

The self-assembly of proteins and peptides into amyloid-like fibrils is mainly known for its notorious role in many pathological conditions such as Alzheimer's disease, Parkinson's disease, and type II diabetes.^1^ However, protein fibrils are known to play functional roles in many organisms, including bacteria, yeasts and humans.^1,2^ Since amyloid-like fibrils have been found to possess remarkable stability, tensile strength and tunable physicochemical properties, they can be fabricated into smart materials for various bio-nanotechnological applications.^3,4^ Amyloid-based hydrogels (AbHs) are one of such smart materials and are very useful for various biomedical and nano-technological applications.^5–7^

Amyloid-based hydrogels (AbHs) are water-laden, three-dimensional materials formed by cross-linking of protein fibrils. The ability of AbHs to hold large amounts of water comes from the fact that they have a large number of hydrophilic functional groups present in their polymeric chains. They resemble living tissues because of their viscoelasticity and biocompatibility.^8^ They could be very useful in the development of materials for different biomedical applications such as superabsorption, drug delivery and tissue engineering.^5–7,9^ Recently, Yang *et al.* have prepared a low-cost injectable AbH, which can carry embedded drugs.^10^ Moreover, JD *et al.* have developed a stimuli-responsive AbH, which may be suitable as an injectable material for cell delivery and tissue engineering applications.^11^

Several proteins and peptides are known to form hydrogels *via* the cross-linking of amyloid fibrils.^10–13^ The conversion of fibrils into thermo-responsive hydrogels was found to be generally controlled by the concentration of proteins/peptides and the temperature of the solutions. Fmoc-protected amyloid beta peptides form AbH on cooling the heated peptides to room temperature.^14^ Yan *et al.* have shown that adding 20 mM DTT to a 3.0 mM lysozyme solution, heating at 85 °C for 10 min and cooling to room temperature lead to the formation of amyloid-based hydrogels.^15^ Using multiple techniques, they have also demonstrated that the hydrogels were formed *via* network formation of amyloid fibrils during the cooling process.^16^ However, it is not clear how these proteins behave during heating and cooling processes and what is the effect of protein concentrations on the process. Therefore, in this study, we have systematically answered the following questions: (1) can unfolding/refolding, aggregation and cross-linking reactions be studied independently? (2) How does HEWL in the presence of the reducing agent TCEP unfold and refold during heating and cooling processes? (3) What is the conformation of the protein at the heating and cooling end of the above three reactions? and (4) what is the correlation between the formations of the monomeric aggregation precursor state, the amyloid fibrils and hydrogel formation?

In this study, we found that HEWL at protein concentrations ≥300 μM preincubated with TCEP in a TCEP/HEWL molar ratio of 4 : 1 and heated at 90 °C, converted into a self-healing hydrogel immediately after cooling the solution to 25 °C. We also standardized the concentrations of HEWL at which it unfolds and refolds into monomeric states under hydrogel fabrication conditions. We identified the monomeric aggregation precursor (I) state of the lysozyme formed by the refolding of the completely unfolded protein. We characterized the conformation of the I state using multiple spectroscopic techniques and found that it resembled a pre-molten globule state of proteins. We also standardized a range of HEWL concentrations at which it formed amyloid fibrils, but not the hydrogel. We established a correlation between these individual reactions. The combination of these individual steps reveals the global mechanism for the fabrication of this hydrogel. The mechanism provided here involves structures and the basis of the formation of on-pathway intermediate for hydrogel formation. We believe that this study will help in the design of desired hydrogels for various biomedical applications and materials development.

## 2. Experimental section

### 2.1. Materials

Hen egg white lysozyme (HEWL), Congo red (CR), tris(2-carboxyethyl)phosphine (TCEP) and thioflavin T (ThT) were purchased from Sigma Aldrich Co., USA. Rest of the chemicals used during the experiments were of analytical grade with purity greater than 99%. A PICO^+^ Benchtop pH-meter (LabIndia Instruments Pvt Ltd) was used for pH measurements. A 0.2 μm Millex-LG syringe filter (Millipore, USA) was used to filter the buffer solutions before use. The concentrations of HEWL, CR, ANS and ThT solutions were determined using a UV/visible spectrophotometer (UV-1800, Shimadzu Corp, Japan), as described previously.^17^

### 2.2. Preparation of the hydrogel

Hydrogel formation was initiated by a combination of heating and cooling steps. Briefly, samples containing 700 μM of HEWL and various concentrations of TCEP in Tris buffer at pH 7.4 were heated at six different temperatures for a period of 2–10 min in a water bath. The heated samples were brought back to 25 °C and were observed for the hydrogel formation using the test tube inversion test.

### 2.3. Transmission electron microscopy (TEM)

The morphology of the aggregates and hydrogel materials was analysed using transmission electron microscopy (TEM) imaging. The aliquots of five-fold diluted samples were placed on a 200-mesh carbon-coated copper grid. After incubation for 2 min, the samples were stained using a 2% (w/v) uranyl acetate solution for 2 min. Excess stain was removed, and the samples were allowed to air-dry. The samples were analysed utilizing a Philips CM200 TEM operating voltage of 20–200 kV with a resolution of 2.4 Å.

### 2.4. FTIR spectroscopy

FTIR spectra of native HEWL and the hydrogels were scanned between 1700 cm^−1^ and 1600 cm^−1^ (amide I band) using a Vertex-80 FTIR instrument (Bruker, Germany) equipped with a DTGS detector. 10 μl of each sample was placed and dried on a translucent KBr pellet, which was made as described previously.^14^ 10 μl of each blank sample (sample – protein) was also put on another KBr pellet and used for the background spectrum. The spectra were deconvoluted by Fourier self-deconvolution (FSD) method. The deconvoluted spectra were then subjected to the Lorentzian curve fitting procedure using the opus-65 software.

### 2.5. Far-UV circular dichroism (far-UV CD)

Far-UV spectra of the samples were recorded between 250 and 200 nm on a Jasco J-815 spectropolarimeter (Tokyo, Japan). For this purpose, a protein concentration of 5 μM, a 1 mm cuvette and a slit width of 1 nm were used. All the spectra were background subtracted with their respective blank. The data was converted into mean residue ellipticity [*θ*] in deg cm^2^ dmol^−1^ using eqn (1).

1 *θ* = CD/(10 × *l* × *n* × *C*)

In this equation CD is the ellipticity in millidegrees, *n* is the number of amino acid residues, *l* is the path length of the cell in cm, and *C* is the molar concentration of the protein. The contents of α-helices and β-strands of the monomeric states were determined using the K2D3 deconvolution software.^18^

### 2.6. Thioflavin T (ThT) binding assay

For each sample, a fixed ThT/protein molar ratio of 5 : 1 was used for ThT fluorescence measurements. The fluorescence was recorded on a Cary eclipse fluorescence spectrophotometer at 25 °C using a 1 cm path length cuvette. The emission spectra were recorded between 460 and 600 nm by exciting the samples at 444 nm. All the spectra were background subtracted with their respective blank.

### 2.7. Congo red (CR) binding assay

For each sample, a fixed CR/protein molar ratio of 5 : 1 was used for CR difference spectra measurements. After 5 min of equilibration, absorption spectra were acquired from 400 nm to 700 nm. The difference spectra were obtained by subtracting the spectra of Congo red alone and hydrogel/aggregates alone from the individual spectrum of each CR-complex.

### 2.8. Sol–gel transition study

The transition of protein solutions into the gel form was measured by the ball-drop method on a home-made apparatus. A HEWL (700 μM) sample with a TCEP/protein molar ratio of 4 : 1 was prepared and heated to 90 °C for 2 min. A 78 mg steel ball was placed on the surface of the samples using a sliding applicator with a hole in the middle. The sample tube was placed into a glass vessel filled with silicone oil and heated to the desired temperature using a thermo-controlled stirrer. The travel of the ball in the tube was video-recorded at every 5 degree intervals from 90 °C to 20 °C. The accurate time of the fall of the ball over a pre-marked distance was calculated using Filmora9 video-editor (https://filmora.wondershare.com/video-editor/). The data was presented as ball-drop time (BDT) *versus* temperature. The sol–gel transition was also measured by 90° resonance light scattering (RLS), as described in the next section.

The fraction (*f*) of aggregate (*f*_scat_) and gel (*f*_gel_) was calculated from the BDT and RLS data using eqn (2):

2 *f* = (*y* − *y*_sol_)/(*y*_gel_ − *y*_sol_)

where *y*, *y*_sol_ and *y*_gel_ represent the observed BDT or RLS at temperature *T* (°C), of the solution phase and gel phase of HEWL, respectively. The *y*_sol_ and *y*_gel_ values in the transition region were obtained by the linear extrapolation of the signal values observed in the temperature regions before and after the transition. In the case of RLS data, *y*_gel_ was replaced with *y*_scat_.

The transition mid points (*T*_m_) of all the curves were determined using the sigmoidal eqn (3)3
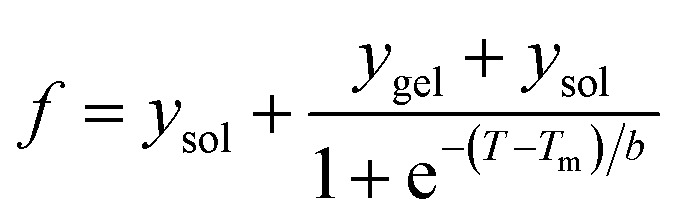
where *b* is the slope factor of the transitions.

### 2.9. Aggregation study

Samples containing different concentrations of HEWL (<300 μM) preincubated with TCEP at a TCEP/protein molar ratio of 4 : 1 were heated at 90 °C for 2 min. The heated samples were cooled at 3–5 degree intervals from 90 °C to 25 °C. At each temperature, the aggregation state was monitored using a 90° resonance light scattering (RLS) assays, as described recently by our group.^19^

The aggregation profile thus generated was fit using the following sigmoidal function (eqn (1)).4
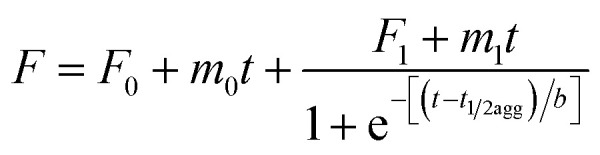
where *F*_0_ and *F* are the observed RLS values at 90 °C and T is temperature in °C. *t*_1/2agg_ is the temperature at which aggregation reaches 50% of the maximum aggregation. *m*_0_ and *m*_1_ are the slopes of the pre- and post-transition regions of the aggregation profiles, respectively. *b* is the slope factor of the transition region.

### 2.10. Unfolding/refolding study

For thermal unfolding, protein samples (3 μM) with a TCEP/protein molar ratio of 4 : 1 were heated from 25 °C to 90 °C. To examine the refolding process, samples were cooled from 90 °C to 25 °C. The process of unfolding and refolding was monitored using intrinsic fluorescence, far-UV CD and ANS fluorescence as previously described by our group.^17,20^ The resulting transition curves were also analysed to calculate the *T*_m_ of unfolding/refolding reactions as previously described by our group.^17,20^

## 3. Results

### 3.1. Sample space of hydrogel formation

**Fig. 1:**
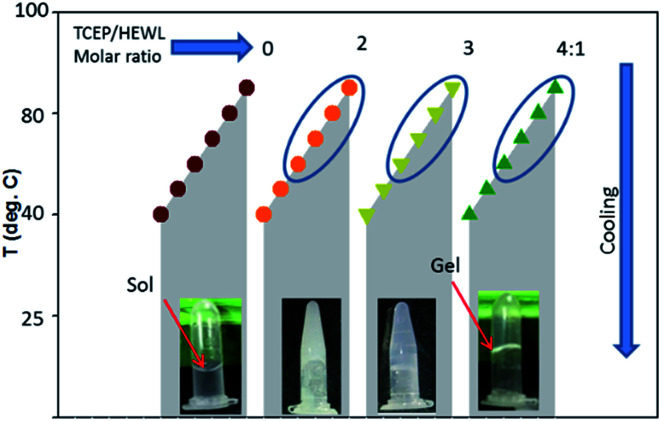
Effect of TCEP concentration and temperature on transparent hydrogel formation by HEWL. Samples containing different TCEP/HEWL molar ratios were heated at various temperatures. Samples inside the blue ellipsoid formed hydrogels on cooling to 25 °C. The protein incubated with a TCEP/HEWL ratio of 4 : 1 formed transparent gel (4^th^ tube from the left), whereas the protein samples without TCEP remained in the solution phase (first tube). All tubes are in inverted position and the red arrows show the location of the menisci between the sol and gel phases.

Hydrogel formation by hen egg white lysozyme (HEWL) was induced by the heating/cooling processes. Fig. 1 shows the schematic of the sample space for the formation of the lysozyme hydrogel (H). First, HEWL (700 μM) samples preincubated with different concentrations of TCEP at pH 7.4 were heated at six different temperatures ranging from 40 °C to 90 °C. Second, these samples were allowed to cool to 25 °C. Finally, the hydrogel formation of the protein was examined using the tube inversion test.^21^ Samples with TCEP heated at and above 60 °C were observed to form hydrogels immediately when the temperature was brought down to 25 °C. It was also found that protein solutions preincubated with a TCEP/HEWL molar ratio of 4 : 1 formed transparent gel (Fig. 1). However, the samples incubated with lower molar ratios of TCEP/HEWL converted into turbid gels (Fig. 1). The turbidity of the gels was observed to decrease with the increase in the concentrations of TCEP. HEWL has four intramolecular disulphide bonds, and when it was preincubated with TCEP in fourfold excess, it unfolded completely between 55 °C and 90 °C (as discussed later), indicating that all the disulphide bonds were broken during the heating of the samples. It has also been reported that all four disulphide bonds of HEWL can be reduced by TCEP under partial/full unfolding conditions.^22^ The turbidity of the gel at the lower TCEP/HEWL ratios may be due to disulphide bond scrambling, which is known to promote amorphous aggregation.^23^ From these results, it appears that the precursor state of the gel may be formed from the fully reduced, unfolded state of HEWL.

The effect of protein concentration on hydrogel formation was also studied by cooling the preheated samples (90 °C), containing TCEP/HEWL in a molar ratio of 4 : 1, to 25 °C and using the tube inversion test. It was observed that at least 400 μM HEWL was needed to form a stiff gel. Below 400 μM of HEWL, the stiffness of the hydrogels was found to decrease with the decrease in protein concentrations. Hydrogel formation was not observed at protein concentrations below 300 μM even after extended incubation at 25 °C. We also observed that the gel formed under the above conditions had excellent self-healing properties. The hydrogel was found to reform within few seconds after vortexing the tube for several seconds (https://drive.google.com/file/d/1Ct6eU602Ylxk9qOGQHJlAgzaY_V6G344/view?usp=sharing).

### 3.2. Characterization of the hydrogel constituting material (HCM)

**Fig. 2:**
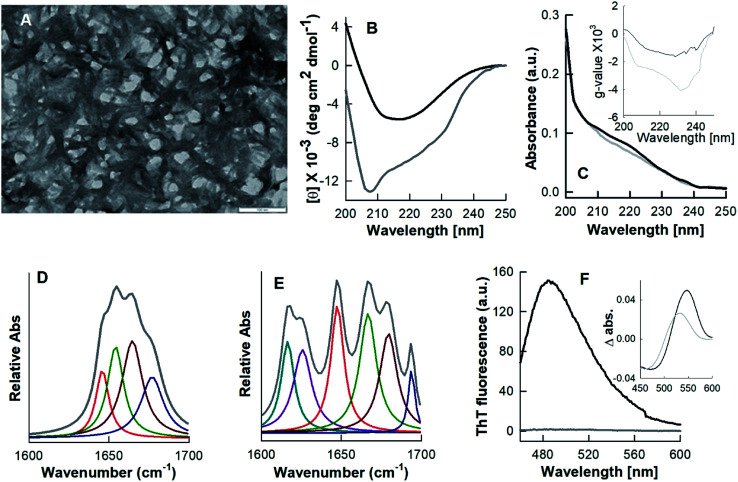
Morphological, structural and tinctorial properties of the hydrogel constituting material (HCM). The transmission electron microscopy image of the negatively stained, fivefold diluted hydrogel (A), far-UV CD spectra of the native protein N (grey curve) and the HCM (black curve) (B), absorbance (C) and *g*-value (inset of C) spectra of native state and HCM. FTIR spectra of the native protein (D) and the HCM (E). The fluorescence spectra of thioflavin T-stained native protein (grey curve) and HCM (black curve) (F). The inset of F shows the Congo red difference spectra of N (grey curve) and the HCM (black curve). The scale bar in (A) represents 100 nm.

To explore the nature of the cross-linked material constituting the HEWL hydrogel, the morphology of the HCM was determined by transmission electron microscopy (TEM). Fig. 2A shows the TEM image of the negatively stained hydrogel diluted five-fold in double distilled water. The analysis showed the presence of well-defined fibrillar structures intertwined with each other. The TEM image of a further diluted (tenfold) hydrogel sample showed that the HCM comprises short straight fibrillar structures of around ∼10–20 nm in width and ∼200–400 nm in length (Section 3.4, Fig. 4D). The TEM image of these diluted samples shows the formation of several thinner fibrils or protofilaments associated to each other laterally.

The secondary structures of the native protein and the hydrogel were studied using far-UV CD and FTIR spectroscopy. Fig. 2B shows the far-UV CD spectra of the native state and HCM. The spectrum of native HEWL displays strong negative bands in the range from 200 to 230 nm, and its negative ellipticity at 208 nm is greater than that at 222 nm (Fig. 2C), which is characteristic of an α + β protein.^24^ The far-UV CD spectrum of the HCM indicates the presence of mainly an extended β-sheet conformation, as revealed by the single negative band at 215–218 nm.^24,25^ The far-UV CD data were also analysed by calculating Kuhn's *g*-value, which is the ratio of a sample's CD and absorbance.^26,27^ The *g*-value is an intensive property independent of the path length and concentration of the protein. The changes in *g*-values of spectra provide information about the change in the secondary structure of the protein. The absorbance and CD values were measured, as described in previous reports.^26,27^Fig. 2C and the inset of Fig. 2C show the absorbance and *g*-value spectra of the native form and HCM of lysozyme. The *g*-value spectra of N and HCM are similar to those of α + β and all-β proteins.^26^ This also indicates that the HCM is mainly in beta sheet conformation. Far-UV CD seems to be less reliable for the determination of the secondary structure contents of aggregated proteins. Therefore, secondary structure contents of the native state and the HCM were investigated by FTIR spectroscopy. Fig. 2D and E show the FTIR spectra of the native protein and the HCM in the amide 1 region.

Amide I peak deconvolution of native protein using Fourier self-deconvolution (FSD) method and Lorentzian curve fitting procedure using the opus-65 software shows a secondary structure composition of 46% α-helices, 20% β-sheets and 34% turns and random structures. These values are in agreement with X-ray crystallography data.^28^ However, the hydrogel has 53% β-sheets, 25% β-turns and 22% random structures. The peak at 1654 cm^−1^ corresponding to the α-helices of the native protein is not observed in the hydrogel spectrum. Thus, the material of hydrogel has dominant β-sheet structures and contains bands at 1616 and 1626 cm^−1^, which are considered diagnostic for the β-sheet formation associated with amyloid fibrils formation.^29^

Fig. 2F shows the fluorescence spectra of ThT-dyed native protein and HCM. The ThT fluorescence spectrum of the gel exhibits the characteristic peak of an amyloid fibril-ThT complex at 485 nm, whereas the native state does not show any significant ThT fluorescence. We also examined the samples with the Congo red (CR) binding assay. The CR difference spectrum of hydrogel reveals a maximum at 540 nm (inset of Fig. 2F). Similar CR difference spectra have been observed for amyloid-like fibrils for many proteins.^17,19,30,31^ ThT and CR interact with the cross-β motif and give information about the secondary structure of the samples.^17,19,31,32^ The ThT fluorescence and CR difference spectra analyses showed a marked ability of the hydrogel to interact with these dyes and cause the spectral changes associated with amyloid fibrils. The high content of β-sheet structures observed in the HCM is in agreement with the ability of the same samples to bind amyloid-diagnostic dyes, which suggests that such β-sheet structures are mainly intermolecular.

### 3.3. Temperature dependence of sol–gel transitions

**Fig. 3:**
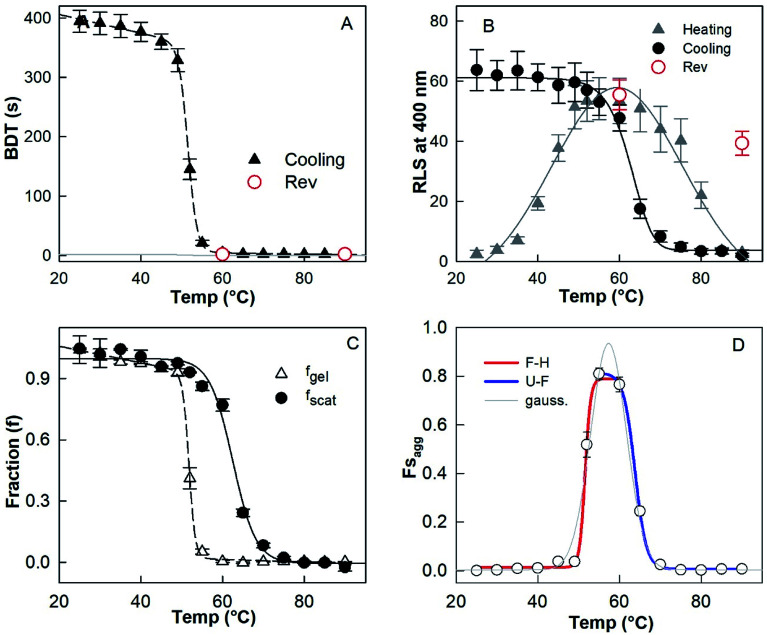
Effect of temperature on the transition of solution phase to gel phase as monitored by ball drop time (BDT) (A). The effect of heating and cooling of the sample as monitored by RLS. The sample contains 700 μM protein in the presence of a TCEP/protein molar ratio of 4 : 1 at pH 7.4 (B). The gel fraction *f*_gel_ and aggregates fraction *f*_scat_ in the sol and gel phases are calculated by using eqn (3) (C). Population curve of the fraction of aggregate in solution phase; U represents the monomeric unfolded state, F is the fibril state and H is the gel state (D).

Fig. 3 shows the cooling-induced transition of solution-state (sol) HEWL (700 μM) into gel state (gel) at pH 7.4 and 90 °C in the presence of a TCEP/protein molar ratio of 4 : 1. The sol–gel transition was monitored by the ball drop method (Fig. 3A) and 90° resonance light scattering (RLS) (Fig. 3B). The ball drop time (BDT), the time of travel of the ball between two points through the samples, and RLS values provide information about the rheological (flow) properties and aggregation of the protein, respectively.^17,18,33^ The transitions monitored by BDT and RLS were found not to be superimposable, when normalised to the gel fraction (*f*_gel_) and the aggregate fraction (*f*_scat_) (Fig. 3C). This indicates that the formation of the aggregate and its cross-linking to form the gel are independent reactions. The aggregation reaction precedes the gelation reaction. We calculated and plotted the difference of *f*_scat_ and *f*_gel_, which gave a fraction of aggregate in solution phase (*f*s_agg_) population curve against temperature. This population curve gives information about the formation of *f*s_agg_ and its subsequent incorporation into the hydrogel during the cooling process. As can be seen from Fig. 3D, Fs_agg_ increased below 70 °C and reached a maximum value at 57.3 °C. Below 57 °C, *f*s_agg_ decreased and approached zero at ∼45 °C. The temperature (57.3 °C) at which *f*s_agg_ showed its maximum value was calculated by fitting the data in Fig. 3D using a Gaussian function. Moreover, it was also observed that the RLS values of the sample at 80–90 °C were comparable to those for the native protein at identical concentrations and at the same temperature, which is only slightly higher than the buffer solution at pH 7.4. These results indicate that HEWL, in solutions with a TCEP/HEWL molar ratio of 4 : 1, exist essentially in a monomeric unfolded state (U) between 90 °C and 75 °C, a fibril state (F) around 57 °C and a gel state (H) between 45 °C and 25 °C. Moreover, the decrease of Fs_agg_ below 57 °C suggests that the hydrogel forms at the expense of Fs_agg_. We also examined the reversibility of U to F and F to H transitions by re-heating the H at 60 °C and 90 °C (Fig. 3A and B, open red circles). We found that H completely converted into the solution phase at both temperatures. Instead, this reversibility monitored by RLS showed that the sample retained almost all F form at 60 °C and only partially disaggregated at 90 °C. This data suggests that the F–H transition is reversible, whereas the U–F transition is not reversible. On the basis of these results, it appears that the formation of the H state from the U state is a two-steps and three-states reaction, and the mechanism can be represented as follows (eqn (5)).

5 U → F ↔ H

In Fig. 3D, the region between temperature 90 °C and 55 °C showed the HEWL aggregation profile, whereas data between 55 °C and 25 °C corresponded to the F–H transition. Moreover, the data obtained by BDT also represented the F–H transition. All the three profiles satisfactorily fit (*R*^2^ = 0.994–0.997) by sigmoidal function fit (eqn (3)) and provide information about the midpoints of U–F and F–H transitions. The midpoints of U–F and F–H transitions were thus calculated to be 63.3 ± 4.5 °C and 51.5 ± 3.8 °C respectively, from population curves data (Fig. 3D). The midpoint of the F–H transition as calculated from BDT data was found to be 51.6 ± 3.5 °C, which is almost identical to the midpoint of the same transition calculated from the population diagram.

### 3.4. Effect of protein concentration on the U–F transition

**Fig. 4:**
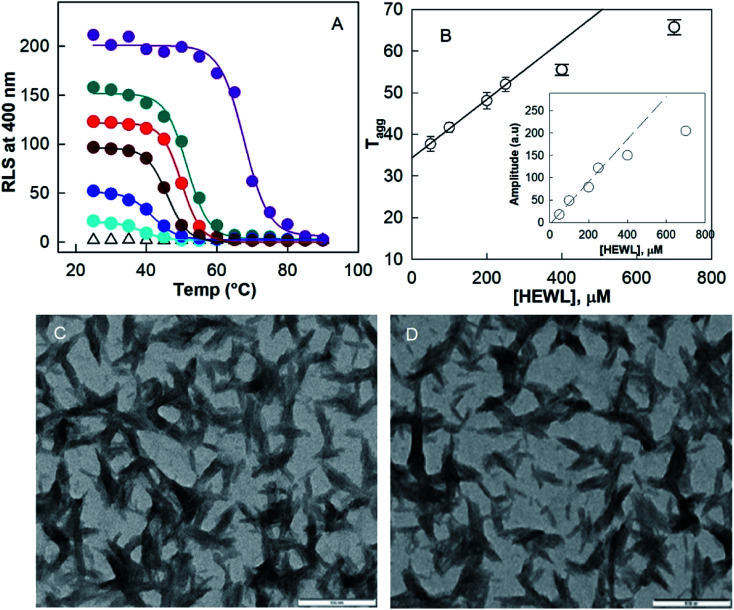
The effect of protein concentration on the aggregation of HEWL during cooling the protein sample from 90 °C to 25 °C in the presence of a TCEP/HEWL molar ratio of 4 : 1 (A). Aggregation was monitored by RLS at 400 nm. Effect of protein concentration on midpoint (B) and amplitude of the aggregation (inset of B) as calculated by fitting the data in (A) using eqn (3). TEM image of the aggregate formed by a 250 μM protein solution under the above solution conditions (C) and the diluted hydrogel (D). The scale bar represents 100 nm.

Since HEWL did not form a hydrogel at protein concentrations below 300 μM, we studied the effect of protein concentration on U–F transitions using RLS at 400 nm. To see how the U–F transition is affected under gelation conditions, we also examined the same aggregation process at protein concentrations of 400 and 700 μM. Fig. 4 shows the effect of protein concentration on the aggregation of HEWL during cooling of the protein sample from 90 °C to 25 °C in the presence of a TCEP/HEWL molar ratio of 4 : 1. Fitting the data with a sigmoidal function (eqn (3)) gave parameters such as the amplitude (*A*) and the midpoint temperature (*T*_m_ = *T*_agg_) of the aggregation process. *T*_agg_ and *A* give information about the temperature at which 50% aggregation is completed and the amount of aggregates at the end of the reaction, respectively. Both the parameters, *T*_agg_ and *A*, were found to increase linearly up to 250 μM protein concentration, whereas they markedly deviated downward from linearity at the protein concentrations at which gelation occurs *i.e.* 400 and 700 μM of HEWL. The extrapolated line fit to the data up to 250 μM concentration intercepted the *x*-axis at 10.3 μM of HEWL. This indicates that during the F–H transition, the determination of *A* or *T*_agg_ may be underestimated. Moreover, the occurrence of zero amplitude at concentrations of protein ≤10.3 μM suggests that the protein did not aggregate below 10 μM.

Fig. 4C shows the TEM images of aggregates formed on cooling the preheated sample from 90 °C to 25 °C at 250 μM HEWL. It can be noted that the diluted hydrogel (Fig. 4D) and the aggregates formed at non-gelation concentrations of the protein have very similar morphology. Both contain short fibrils of width ∼20 nm and length ∼200–400 nm. These results suggest that fibrils formed under either gelation or aggregation concentrations of the protein are similar in morphology. It is very likely that fibrils formed under aggregation conditions are on-pathway intermediates of H formation.

### 3.5. Unfolded HEWL converted into an intermediate state upon cooling

**Fig. 5:**
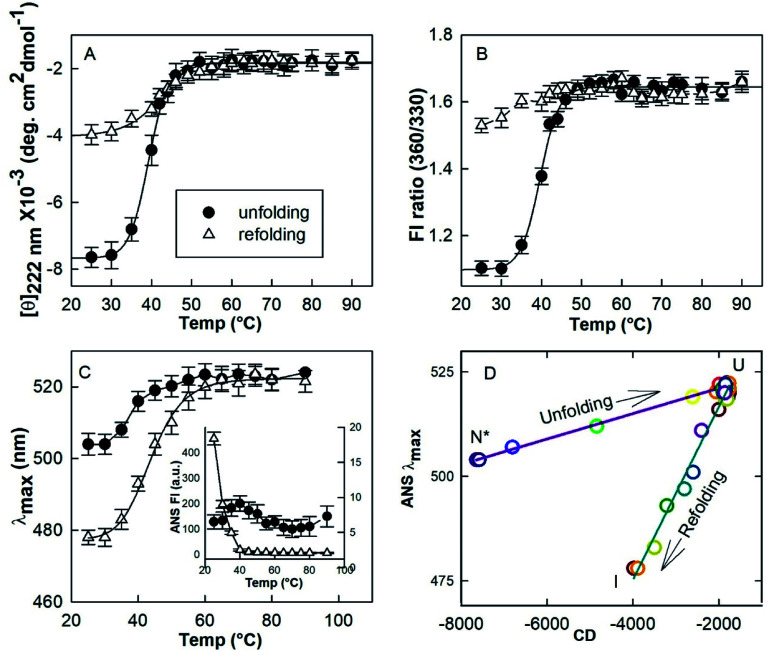
Temperature induced unfolding and refolding of HEWL in the presence of a TCEP/HEWL molar ratio of 4 : 1. The unfolding/refolding transition was monitored by far-UV CD at 222 nm (A), the ratio of fluorescence intensities FI 360/330 (B) and the maximum emission (*λ*_max_) of ANS fluorescence (C). The phase diagram separating native-like (N*), unfolded (U) and refolded state (I) was generated by plotting *λ*_max_ of ANS fluorescence *versus* mean residue ellipticity at 222 nm (D).

Since HEWL did not aggregate below 10 μM protein concentrations (Section 3.4) neither during heating or cooling, temperature induced unfolding and refolding of HEWL (4 μM) in the presence of a TCEP/protein molar ratio of 4 : 1 at pH 7.4 was followed using various spectroscopic techniques. Mean residue ellipticity at 222 nm ([*θ*]_222 nm_) was used to probe the nature of the secondary structure (Fig. 5A).^34^ The changes in tertiary conformation were monitored by fluorescence intensities at 360 nm and 330 nm (FI ratio 360/330) (Fig. 5B). The FI ratio 360/330 gives information about the extent of solvation of the protein core and is used as a probe of the tertiary structure.^17,35^ Maximum emission wavelength (*λ*_max_) of ANS fluorescence was used to monitor the changes in exposed hydrophobic clusters.^36^ All probes show that HEWL in the presence of a TCEP/HEWL molar ratio of 4 : 1 unfolds in the 35–50 °C temperature range. All the transitions fit satisfactorily (*R*^2^ = 0.992–0.997) with the two-state transition model. The midpoints of thermal transition (*T*_m_) were calculated to be 38.8 ± 1.6, 39.6 ± 2.2, and 39.1 ± 2.3 for [*θ*]_222 nm_, FI ratio 360/330 and *λ*_max_ of ANS fluorescence data, respectively. It was also found that the ANS fluorescence intensity remained low and almost constant (6–9 a.u.) in the transition region. It has been reported that ANS emission intensity increases and *λ*_max_ shifts to shorter wavelengths when ANS interacts with certain intermediate states of a protein.^36,37^ These observations suggest that the observed transition has occurred between two conformational states *viz*. N* and U, which exist between 25–30 °C and 60–90 °C, respectively.

In order to understand the initial conformation of the material composing the hydrogel, the effect of cooling was investigated by using all the three probing techniques mentioned above. It was found that the protein retains its U state between 90–60 °C on cooling. However, the negative [*θ*]_222 nm_ increases from ∼1900 deg cm^2^ dmol^−1^ at 90 °C to ∼4000 deg cm^2^ dmol^−1^ at 25 °C. FI ratio 360/330 decreased from ∼1.6 to ∼1.4. The *λ*_max_ of the ANS emission shifted from 523 nm to 478 nm. An increase close to 95-fold in the ANS intensity was observed when the sample was cooled to 25 °C from 90 °C. These results suggest that U converts into a partially folded intermediate (I) on cooling.

### 3.6. The partially folded intermediate resembles a pre-molten globule state

**Fig. 6:**
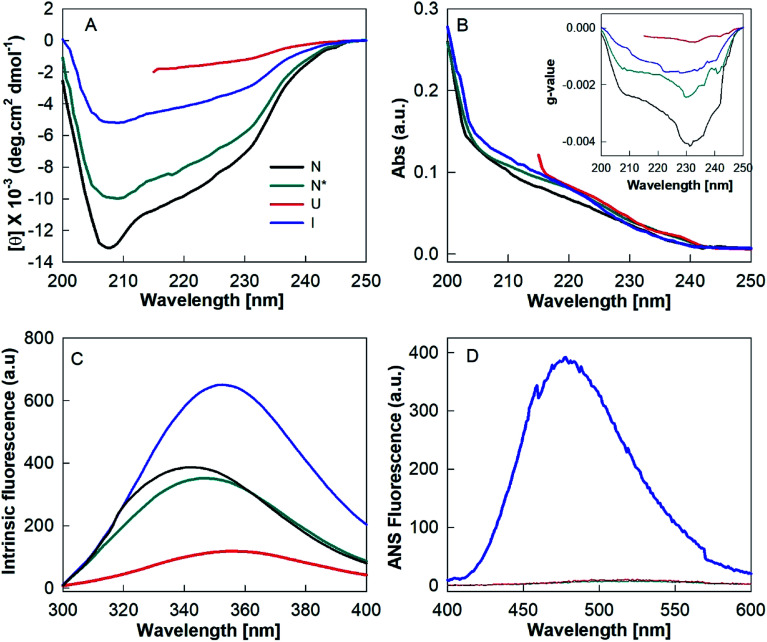
Structural properties of different states of hen egg white lysozyme. Far-UV CD (A), absorbance (B), *g*-value (inset B), intrinsic fluorescence (C) and ANS fluorescence (D) spectra of native (N), TCEP-induced (N*), partially folded (I) and unfolded states of lysozyme.

The biophysical properties of N*, U and I states were examined by various probing techniques for secondary structure, tertiary structure and exposure of hydrophobic clusters at their surface. Fig. 6A shows the far-UV CD spectra of these states along with the spectrum of native HEWL at pH 7.4 (N). The far-UV CD spectrum of the N state is typical of a folded α + β protein with the negative ellipticity at 208 nm greater than that at 222 nm.^24^ However, the decrease in negative ellipticity values for the N* state suggested that a significant amount of the secondary structure was disrupted during the N–N* transition. The spectrum of the HEWL solution with a TCEP/HEWL molar ratio of 4 : 1 at 90 °C (U) (Fig. 6A, red curve) is almost identical to the completely unfolded state of HEWL induced by 6 M guanidine hydrochloride and 3 mM TCEP (U_G_, spectrum not shown). This indicates that U is a random coil state without any significant residual secondary structure.^24^ The spectrum of the I state of HEWL, which accumulated by cooling of the U state to 25 °C, showed significantly negative ellipticity values between 208 and 222 nm compared to those of the U state. This indicates that the I state contains a significant amount of secondary structures. Fig. 6B and its inset show the absorbance and *g*-value spectra of N, N*, I and U states of the lysozyme. The *g*-value spectra also showed significant changes in secondary structure between different states of the protein. The higher negative *g*-value of the I state as compared to that of the U state again suggested the presence of some residual secondary structures.

The amount of regular secondary structures *viz*. α-helices and β-strands for N, N* and I states were calculated by analysis of the CD spectra using the K2D3 deconvolution software.^18^ The secondary structural contents (α-helices and β-strands) of these states are given in Table 1. These analyses indicate that the I state, the precursor state of the fibrillar material of the hydrogel, contains around 8.6% α-helices and 26.7% β-strands. Since the occurrence of these beta structures in the I state is significantly higher (∼111%) than in the N state (12.6%), it is highly probable that the secondary structures of the I state are non-native in origin.

**Table 1:** Conformational properties of different states of HEWL accumulated during hydrogel formation

Protein states	K2D3		Intrinsic fluorescence		ANS fluorescence	
	% α-helices	% β-stands	*λ*_max_	FI at *λ*_max_	*λ*_max_	FI at *λ*_max_
N	39.2 ± 2.8	16.6 ± 1.5	343.9 ± 1.2	386 ± 10	524.5 ± 1.7	6.8 ± 1.1
N*	29.8 ± 2.3	14.8 ± 1.3	347.5 ± 1.0	351 ± 08	522.6 ± 1.4	8.7 ± 1.2
I	8.6 ± 1.1	26.7 ± 2.2	352.6 ± 1.2	650 ± 11	477.2 ± 1.2	391.6 ± 9.8
U	—	—	355.5 ± 1.4	118 ± 07	524.7 ± 1.6	6.9 ± 1.8
UG	—	—	356.2 ± 1.5	175 ± 06	525.0 ± 1.3	5.8 ± 1.6

Fig. 6C shows the intrinsic fluorescence spectra of N, N*, U and I states of HEWL. As presented in Table 1, N* and U states of HEWL showed a red shift of ∼3.6 nm and ∼9 nm and a decrease of ∼9% and ∼69% in fluorescence intensity at 340 nm, respectively, as compared to those of the N state. However, U_G_ and I states showed a red shift of ∼10 nm with ∼55% decrease and 68% increase in the fluorescence intensity, respectively, as compared to that of N state (Table 1). These results indicate that during the N–N* transition, Trp residues of the protein were only partially exposed to the solvent. In U and I states, Trp residues were found to be fully exposed to the solvent suggesting the absence of intact tertiary structures in both U and I states. However, differences in intensity compared to N indicate that the unfolded conformations of U and I are different in nature.

The changes in ANS fluorescence spectral features such as *λ*_max_ and emission intensity upon binding with a protein molecule provide information about the protein's conformational states.^36–38^ ANS preferentially interacts with partially folded intermediate states, causes a blue shift in *λ*_max_ and a marked increase in emission intensity compared to the relative aqueous solution.^36–38^ ANS is a hydrophobic dye and strongly binds to hydrophobic clusters, which are exposed to the solvent in the PFI states. However, ANS does not bind, or binds weakly with, the native or unfolded states of a protein, because the hydrophobic clusters are generally buried in the native state and completely disrupted in the unfolded state.^39^ We found that only the I state of HEWL produced spectral changes in ANS fluorescence, meaning it corresponds to an intermediate state (Fig. 6D and Table 1). The enhanced ANS fluorescence and the blue-shift from 526 to 478 nm of the I state compared to the U state suggest that HEWL had also formed solvent-exposed hydrophobic clusters during the cooling process.

## 4. Discussion

**Fig. 7:**
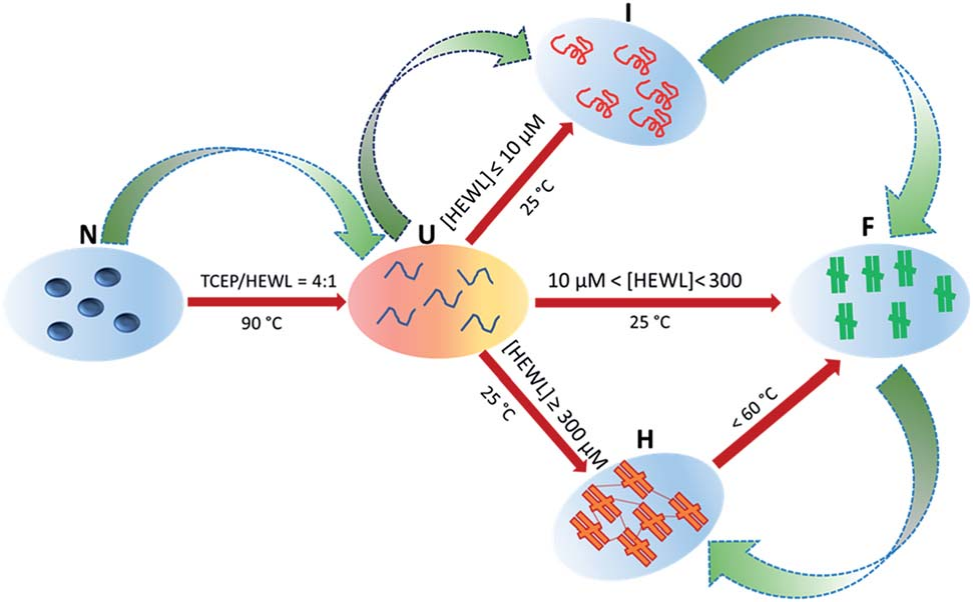
Schematic diagram of heating- and cooling-induced transitions between HEWL states, and subsequent formation of the hydrogel. The N–U transition is common in all reactions. Straight arrows represent the individual reactions of formation of the monomeric aggregation precursor (I), amyloid-like fibrils (F) and the hydrogel (H). Green broad arrows show the overall pathway of formation of the hydrogel.

In this work we have attempted to understand the rationale of amyloid-based hydrogel formation by investigating the temperature- and protein concentration-induced state transitions for HEWL. As depicted in Fig. 7, the native state (N) of HEWL at all tested concentrations appears to convert into an unfolded state at 90 °C in the presence of TCEP/HEWL molar ratio of 4 : 1. At low protein concentrations (<10 μM), HEWL was found to transform into the U state through a two-steps mechanism as shown by the phase diagram in Fig. 5D. The RLS values of the U state, even at higher concentrations, were found to be comparable to those corresponding to identical concentrations of the N state. This suggested that the U state, which exists between 90 °C and 75 °C at all the tested concentrations of HEWL, is essentially a non-aggregate. Interestingly, the U state was found to form the partially folded intermediate state (I) at low concentrations (<10 μM), amyloid-like short fibrils (F) at medium concentrations (10μM < HEWL > 300 μM) and a self-healing hydrogel (H) at high (>300 μM) concentrations of HEWL on cooling to 25 °C. However, the hydrogel formation occurred through an aggregate intermediate state, which occurred maximally around 55–60 °C.

The I state is characterized by residual secondary structures, the absence of tertiary structures and the presence of significant amounts of solvent-exposed surface hydrophobic sites. All these three properties of I are essential characteristics of a pre-molten globule state (PMG).^19,39–43^ Due to the ease of structural rearrangement and the presence of sufficient surface-exposed hydrophobic clusters, the PMG state is considered as the most preferred equilibrium intermediate for amyloid fibrils formation.^4,41^ Both globular and intrinsically disordered proteins have been observed to form amyloid fibrils through the PMG state.^19,39–43^ Therefore, it appears that the I state is the aggregation precursor state for HEWL under the current study conditions.

The aggregate formed under identical conditions, but at medium protein concentrations, possessed all the characteristic properties of amyloid fibrils.^1,17,19,44,45^ However, the amyloid fibrils observed here are shorter in length than the typical fibrils of lysozymes.^1,17,19,45^ These fibrils appear to contain several thinner protofilaments associated with each other laterally. Fibrils formed by lateral association of protofilaments are very common in the lysozyme family.^17,19,45^ Interestingly, hydrogel constituting materials formed at high protein concentrations are very similar in morphology to the amyloid-like fibrils formed at medium protein concentrations under identical conditions. From a close look at the TEM images, secondary structures and tinctorial properties, it appears that the F state rearranges into the HCM without much modifications. Moreover, the reversible nature of the hydrogel with temperature suggests that cross-linking is mainly physical in nature. The extremely high self-healing property of the hydrogel may be due short length and lateral association of several constituting protofilaments. Short filaments may cross-link and delink easily without significant breakage. However, larger fibrils are more prone to breakage. The detailed investigation of the mechanism of self-healing property of this hydrogel is out of the scope of this work.

**Fig. 8:**
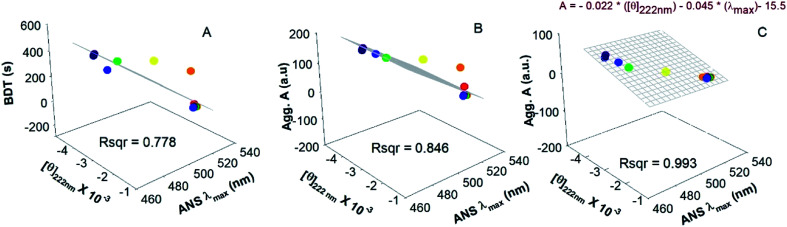
Correlation of properties for the formation of the aggregation precursor state, amyloid fibril and hydrogel. 3D graph showing mean residue ellipticity at 222 nm (*x*-axis), ANS *λ*_max_ (*y*-axis) and RLS values (*z*-axis) for fibril formation (A), for hydrogel formation (B) and BDT (*z*-axis) for hydrogel formation (C). Colored symbols represents data points at different temperatures. The grid represents the plane of best fit to all the data points and corresponds to eqn (6).

In order to understand the connection between I, F and H states of HEWL, we established a correlation between the conformational properties of I, such as secondary structure and surface-exposed hydrophobicity, with F formation and H formation measured by RLS and BDT. Fig. 8 shows three-dimensional graphs obtained by plotting the conformational properties of the I state *viz*. mean residue ellipticity at 222 nm (*x*-axis), ANS *λ*_max_ (*y*-axis) and RLS (*z*-axis) for F formation, H formation and BDT (*z*-axis) for H formation at *z* axis. The data points were fitted with a plane using eqn (6).

6 *z* = *ax* + *by* + *y*0

where *a* and *b* are constants and determine the slope and *y*0 is an *x*–*y* intercept.

We found that the data best fit in case of F formation with *R*^2^ > 0.99 and fairly fit for H formation measured by RLS. The correlations are described by the following equations.

7 RLS = −0.02[*θ*_222 nm_] − 0.04(*λ*_max_) − 15.5

8 RLS = −0.005[*θ*_222 nm_] − 3.5(*λ*_max_) + 1610

However, H formation measured by BDT does not fit (*R*^2^ = 0.667) with the plane equation. These correlations further support our conclusion that hydrogel formation by HEWL involves the I state as a precursor of the HCM, which physically cross-links and forms the hydrogel. Furthermore, the I state controls the formation of the HCM, whereas cross-linking of HCM into H is an independent process.

## 5. Conclusion

We have demonstrated that the individual reactions of a partially folded intermediate state, amyloid-like fibrils and an aggregated state during hydrogel formation by HEWL under identical solution conditions, but different protein concentrations, can gave rise to the global molecular mechanism of hydrogel fabrication. This conclusion cannot rule out some effects of protein concentration on the individual reactions. But given the difficulty in identification and characterization of the intermediates in the pathway of hydrogel fabrication due to high protein concentrations, this study provides a remarkable way to understand the mechanism of hydrogel fabrication. The difference in the structures of partially folded intermediates of a protein is known to generate polymorphism of amyloid fibrils.^46,47^ Therefore, the understanding of “on pathway” intermediates of hydrogel formation can be exploited to engineer the properties of hydrogel for various biomedical applications.

## Conflicts of interest

There are no conflicts to declare.

## Supplementary Material
